# Editorialets

**Published:** 1907-02

**Authors:** 


### Editorialets.

"I ENJOY reading the "Red Back" more than any of the medical journals on my list. C. B. RAINES, M. D., Mineral Wells, Texas."

CASES of lateral curvature of the spine must be early recognized and treated, for otherwise the deformity is apt to become so fixed that a cure is no longer possible.—*International Journal of Surgery.*

DR. HOLMAN TAYLOR of Marshall, Texas, one of the ablest and most popular and zealous of the younger physicians of Texas, has been appointed Assistant State Health Officer. Good. A most excellent selection.

EPILATION is of great importance in all follicular inflammations of the skin, since it not only removes a host of microbes but also affords access for antiseptics to the deeper parts of the follicle.—*International Journal of Surgery.*

NOTICE.—Young physician, two years' hospital experience, by May 1st to 15th, desires to associate himself with established man who is doing some surgery. Address, "Hospital," care TEXAS MEDICAL JOURNAL, Austin, Texas.

THE administration of thyroid extracts or iodides and the application of iodine ointments are only of value in recent cases of soft or parenchymatous goitres, especially in young persons.—*International Journal of Surgery.*

WANTED.—Location in Texas or New Mexico by an eye and ear specialist and expert refractionist. Will sell or exchange fine home and sanitarium for farm or town property in the South. Address, J. L. SHORT, M. D., Versailles, Mo.

DR. S. EAGON of Dallas, the Nestor of the profession of Texas, renews his subscription to the "Red Back" for the twenty-fifth consecutive year, paying five years in advance (to January, 1911), and says: "I am much gratified to see the JOURNAL maintain the very high standard of the past. It is still 'The Little Gem.' "

AMONG the doctors from Texas doing post-graduate work the following are attending the New Orleans Polyclinic, which is now the Post-Graduate Department of Tulane Medical College: Dr. J. M. Andrews, Farmersville; Dr. C. C. Sorrells, Royse City; Dr. J. H. Hendrix, Edgewood; Dr. W. G. Allen, Harleton; Dr. T. G. Boorman, Princeton; Dr. J. A. R. Moseley, Jefferson; Dr. G. Bartlett, Shafter; Dr. W. G. Snipes, Ladonia; Dr. W. O. Warren, Pecan Gap; Dr. G. M. Van Ness, Prairie Lea; Dr. M. C. Bell, Silverton; Dr. H. T. Fry, Wills Point.

PURE FOOD.—Timeliness of interest, aside from any other condition, lends especial importance to the announcement of the early publication of "Foods and Their Adulterations," by Harvey W. Wiley, M. D., to be immediately followed by a companion volume, "Beverages and Their Adulterations." Dr. Wiley is Chief Chemist to the United States Department of Agriculture, at Washington, and his wide researches in the interests of purity in food commodities give anything he might write on the subject an authoritativeness that is unquestioned. The fact that the new National Food and Drugs Law becomes effective after January 1st, and that public interest in it is now at white heat, will no doubt result in quite a demand for both volumes. The books will be generously illustrated from original photographs and drawings.

---

DR. DANIEL'S NEW BOOK.

NEW YORK CITY, January 16, 1907.

DEAR DOCTOR DANIEL: I procured your book, "The Strange Case of Dr. Bruno," and read it at one sitting. Jules Verne has always possessed a charm for me, but you have more science, a more intricate, interest-compelling plot and more marked dramatic style. * * *

You have made a notable contribution to fiction, and I congratulate you. Truly yours,

R. W. WILCOX.

(Dr. Wilcox is Professor of Medicine in the New York Post-Graduate Medical School and Hospital.—D.)

"A stranger story was never penned."—*N. Y. Commercial.*

"One of the literary sensations of the year; a most remarkable book."—*N. Y. Evening Journal and American.*

TOBIN BOOK CO., Austin, Texas. (See Adv.)

---

FOR SALE.—Unopposed practice in small town on Rock Island railroad in North Texas, free to purchaser of good house, lot, etc. Collections 98 per cent. $1000 cash. Address, "T," care "Red Back," Austin, Texas.

---

MARRIED.—At McKinney, Texas, February 7th, Dr. Lewis A. Kirk of Austin, to Miss Helen MacDonald of McKinney.

---

MONTEREY, January 28, 1907.

DEAR DOCTOR DANIEL: Our meeting in Mexico City was a success in every way, and you missed a good thing; every doctor present enjoyed the meeting and was well repaid for the time and expense.

The following officers were elected for the ensuing year: President, Dr. H. V. Jackson, Durango, Dgo.; First Vice-President, Dr. S. Ulfelder, Benito, Juaraz, No. 4, Mexico, D. F.; Second Vice-President, Dr. G. B. Hyde, Silao, Gto.; Secretary and Treasurer, Dr. J. S. Steele, Apt. 227, Monterey, N. L.; Censors, Dr. L. H. Barry, Durango, Dgo. (elected last year); Dr. C. E. Husk, Sta.

Barbara, Chih. (for three-year term); Dr. A. H. Whatley, Parral, Chih. (in place of Dr. Hodson, resigned); Editors of the *Annual,* Dr. R. D. Robinson, Torreon, Coah.; Dr. W. R. Jamieson, Torreon, Coah.

The next meeting will be in Monterey next November. Keep this in mind and help us make it a banner meeting by your attendance and wisdom.

H. V. JACKSON, President,
J. S. STEELE, Secretary.

